# Household firearm access, storage, perceptions, and discussions among U.S. military veterans: population-based estimates from the 2022 ASCEND survey

**DOI:** 10.1186/s40621-026-00692-3

**Published:** 2026-05-31

**Authors:** Lindsey L. Monteith, Joseph A. Simonetti, Julie A. Kittel, Ryan Holliday, Sean M. Barnes, Evan R. Polzer, Claire A. Hoffmire

**Affiliations:** 1 Spark M. Matsunaga VA Medical Center, VA Pacific Islands Healthcare System, 459 Patterson Rd, Honolulu, HI 96819 United States of America; 2 Department of Physical Medicine and Rehabilitation, University of Colorado Anschutz Medical Campus, Aurora, CO United States of America; 3 Department of Psychiatry, University of Colorado Anschutz Medical Campus, Aurora, CO United States of America; 4 Firearm Injury Prevention Initiative, University of Colorado Anschutz School of Medicine, Aurora, CO United States of America; 5 VA Rocky Mountain Mental Illness Research, Education and Clinical Center for Suicide Prevention, Aurora, CO United States of America

**Keywords:** Veteran, Firearm, Lethal means safety, Storage

## Abstract

**Background:**

Firearm injury remains the leading method of suicide among U.S. veterans. Knowledge of the prevalence of firearm access, storage, and beliefs about firearms and suicide risk among veterans is necessary to inform firearm-related suicide prevention initiatives.

**Methods:**

A 2022 population-based survey of veterans (ASCEND), with responses weighted to the veteran population.

**Results:**

In 2022, 57.4% (95% CI: 56.4–58.4) of veterans had access to firearms in their home; of those, 53.7% (95% CI: 52.3–55.1) stored at least some household firearms unlocked and 49.0% (95% CI: 47.6–50.4) stored at least some firearms loaded with ammunition. The most common reason for having household firearms was protection against people (74.3%; 95% CI: 73.2–75.3). The majority of veterans reported agreement with beliefs regarding firearm access impacting suicide risk (e.g., that having a firearm in the home increases risk; 55.2%–71.0%) and indicated willingness to alter their access if thinking about suicide (74.9%–84.6%). Veterans reported the most willingness to discuss firearms with a family member (88.4% willing; 95% CI: 87.7–89.1), compared to friends (84.0%; 95% CI: 83.1–84.7) or providers (72.4%; 95% CI: 71.4–73.4). Although most were willing to discuss firearms with a primary care or mental health provider, only 12.9% (95% CI: 12.1–13.6) indicated that a healthcare provider had ever asked them about their firearm access. Firearm risk perceptions, willingness to change firearm access and discuss firearms, and prior discussions with a healthcare provider differed based on household firearm access responses: those who declined to respond (7.7%; 95% CI: 7.2–8.4) reported the least agreement with firearm risk statements (34.4%–57.7%) and the least willingness to change firearm access (54.5%–73.6%) or discuss firearms with others (75.5% family; 69.0% friends), particularly healthcare providers (35.0%; 95% CI: 31.1–39.0).

**Conclusions:**

More than half of veterans have access to firearms in their home, an important risk factor for suicide. Results suggest a preference to include family in firearm suicide prevention efforts and a need to increase provider firearm discussions in healthcare settings. Additionally, as veterans who decline to respond to firearm access questions appear to differ in their firearm risk perceptions and willingness to reduce firearm access when risk is elevated, better understanding these veterans is essential.

**Supplementary Information:**

The online version contains supplementary material available at 10.1186/s40621-026-00692-3.

## Background

In the United States (U.S.), age-adjusted suicide rates in 2023 were 49.7% higher among males and 103.1% higher among females for veterans, compared to non-veteran adult males and females [1]. Firearm injuries also account for a higher proportion of suicide deaths among veterans, comprising 73.3% of suicide deaths in 2023 versus 52.9% among non-veteran adults [1]. From 2001 to 2023, the firearm suicide rate among U.S. veterans increased by 67.0%, with firearm injury remaining the leading method of suicide throughout this period [1]. Notably, firearm injury is the most lethal method of suicide attempt, with case fatality rates of approximately 90%, exceeding those of other methods [2].

Considering these findings, alongside the strong association between firearm access and suicide risk [3], the U.S. Department of Veterans Affairs (VA) has implemented numerous initiatives to prevent firearm suicide as part of its multi-faceted suicide prevention strategy, which includes numerous lethal means safety (LMS) initiatives aimed at increasing the time it might take for a person with suicidal intent to access lethal methods of suicide [4]. Examples include widespread distribution of cable gun locks to increase secure firearm storage among veterans, distribution of firearm lockboxes to veterans with elevated suicide risk, education and public health messaging campaigns, training healthcare providers in LMS counselling (LMSC), provider screenings of veterans in VA healthcare settings for suicide risk and conducting LMSC when risk is elevated, and partnering with firearm stakeholders to support veterans who decide to store their firearms outside of the home (e.g., firearm retailers; [4–6]). In addition, the VA has invested in research on lethal means of suicide to inform strategies aimed at encouraging veterans to reduce their access to firearms and other lethal methods when suicide risk is elevated [6].

One such initiative is *Assessing Social and Community Environments with National Data (ASCEND) for Veteran Suicide Prevention*, a recurring cross-sectional national survey of U.S. veterans. In addition to increasing knowledge of the prevalence of non-fatal suicidal self-directed violence (NF-SSDV) in the veteran population [7, 8], ASCEND collects information on risk factors for suicide, including firearm access and storage, with item content informed by prior national surveys and refined with the partnership of veterans [9]. Regularly obtaining information on the prevalence of firearm access, firearm storage, and beliefs about firearms and suicide risk among U.S. veterans is crucial to inform national firearm-related suicide prevention efforts for veterans, particularly considering the potential for firearm beliefs and behaviors to change over time, including during, and in response to, national events [10–13].

Prior surveys have reported on the prevalence of personal firearm ownership [14, 15] and storage practices [16, 17] among U.S. veterans, providing a crucial foundation for beginning to understand the extent to which veterans have access to firearms, how they store their firearms, and whether they recognize a relationship between such access and their risk for firearm injury and suicide. In the *2015 National Firearm Survey (NFS)*, which included 1,044 veterans, Cleveland and colleagues found that 44.9% of veterans reported owning a firearm, 3.4% reported being a non-owner living with another firearm owner, and 51.7% neither owned firearms nor lived with a firearm owner [14]. More recently, Fischer and colleagues examined data from the *2022 National Health and Resilience in Veterans Study (NHRVS)*, an anonymous survey drawn from a probability-based survey panel of U.S. adults: 50.9% of veterans reported owning firearms [15].

Both studies also reported on firearm storage practices among firearm owners. In 2015, 66% of veteran firearm owners stored one or more firearms unlocked and 46.7% stored at least one loaded [16]. In the subsequent 2022 NHRVS survey, 52.9% of veteran firearm owners stored personal firearms loaded and/or in a non-secure location, with 39.9% of owners specifically reporting storing at least one personal firearm loaded [17]. These findings are critical for understanding the context of firearm suicide deaths among veterans, although the causal relationship between specific firearm storage practice and suicide risk among adults (particularly firearm owners) remains unclear [18].

Continuing to understand the prevalence of firearm access in the veteran population, as well as factors which may impact veterans’ firearm access and storage decisions (e.g., their perceptions of firearm access as it relates to risk for suicide), remains integral to understanding how to prevent firearm injury and suicide among veterans. The 2015 NFS provided crucial information on the prevalence of household firearm ownership among veterans [14], and the 2022 NHRVS survey provided recent estimates of personal firearm ownership but did not assess household firearm access [15]. Thus, we are unaware of more recent estimates of household firearm access (i.e., the extent to which the veteran has access to firearms within the home, irrespective of whether they own such firearms) in the overall veteran population. This gap is crucial to address considering that household firearm access is associated with increased risk for firearm suicide [3, 19]. As one poignant example, women’s suicide rate increased substantially after another adult with whom they resided became the owner of a handgun, which was specifically due to an increase in the rate of firearm suicide [20]. In fact, specific segments of the veteran population, including women, are less likely to personally own firearms, but are more likely to live in a home with firearms owned by someone else [14, 21, 22]. As women comprise a growing proportion of the veteran population, this is likely to impact veteran suicide rates and population prevalence of household firearm access among veterans over time.

With population-based firearm data among veterans tending to focus on firearm ownership and storage, generalizable information regarding firearm-related risk perceptions and the circumstances in which veterans with firearm access are willing to reduce their access to avert injuries is limited. With only a few exceptions (e.g., [16, 23, 24]), most prior research on these topics has been qualitative in nature and focused on circumscribed samples. Yet understanding veterans’ perceptions of firearms [25], their willingness to change how household firearms are stored, and their preferences for firearm discussions [22, 26–28], is crucial to informing public health messaging campaigns and other LMS efforts (e.g., potential messengers and partners for firearm LMS initiatives).

To address these knowledge gaps and build upon prior research on firearms with U.S. veterans, we analyzed ASCEND (2022) data to examine the prevalence of household firearm access and storage, veterans’ reasons for having household firearms, their perceptions of firearms and suicide risk, willingness to alter their firearm access if thinking about suicide, willingness to have firearm discussions with others, and prior firearm discussion(s) with healthcare providers, in a nationally representative survey of U.S. veterans.

## Methods

### Procedures

Data were collected in 2022 as part of ASCEND Wave 1 (described above; [8]). Study methods have been described in detail previously [8] and pertinent information is summarized below. Wave 1 included two geographically defined samples, including a main sample comprised of veterans in all 50 U.S. states, Washington D.C., and Puerto Rico ([8] the focus of this manuscript) and a separate Pacific Islands Territories pilot sample ([29]; not included in the present analyses). The local IRB and VA Research and Development Committee approved this study. All participants provided informed consent to participate.

### Sampling

The main sample, drawn from the population of U.S. veterans alive in June 2021, used a sampling frame (*N* = 16,738,616 eligible cases) constructed with data from the U.S. Veterans Eligibility Trends and Statistics (USVETS) Database, which provides comprehensive information on all living U.S. veterans, and the VA-DoD Identity Repository (VADIR), a secondary source employed to facilitate comprehensive coverage of recently separated veterans. Exclusion of ineligible records encompassed location (i.e., missing information on state or located in an ineligible state/territory, at a VA facility, military base, or the European Economic Area), an absence of personally identifying information to facilitate contact updating, or designation as deceased or likely not a veteran. Data elements within frame sources were used to assign records to strata and to facilitate stratified sampling, by state, sex, race/ethnicity, and separation date; these elements were used in combination to develop sampling strata, with sampling targets developed around precision goals for specific subdomains and subgroups (e.g., oversampling female and recently separated veterans; [8, 30]. From March through June 2022, 97,287 veterans were invited to participate in the main sample, and 17,396 participated (American Association for Public Opinion Research [AAPOR] [31] of 19.2%; see Supplemental Appendix 1 for additional details).

### Recruitment

Recruitment was sequential, multimode, and included a push-to-web design, which included online (primary), paper (secondary), and computer-assisted telephone interview (available upon request) modes of data collection. Invitations to participate included an initial mailing and email (if available) with a $1 pre-incentive, followed by bi-weekly reminder mailings and subsequent telephone prompts, as well as a mailed paper survey to non-respondents at Week 6. Respondents received a $5 post-incentive, unless declined.

### Measures

Survey content was developed through review of published literature, subject matter expert consultation, and partnership with a project Veterans Engagement Board [8, 9]. The firearm questions (see Supplemental Appendix 2 for item content) assessed household firearm access (i.e., “*Do you have access to firearms in your home*?”). Response options included yes, no, or decline to respond. Respondents who left the question blank (rather than indicating decline to respond) were treated as missing, rather than as declines. Additional items assessed firearm storage, reasons for having household firearms, perceptions of firearms and suicide risk, willingness to alter firearm behaviors if they were thinking about suicide, and whether respondents had ever had a prior discussion with any healthcare provider about firearm access. The survey also asked about veterans’ willingness to discuss household firearm access with various individuals: “*Regardless of whether you have household firearms*,* how willing would you be to discuss your firearm access with each of the following individuals?”* (i.e., primary care or mental health provider; a friend; a family member). Response options were dichotomized as willing (endorsement of “*definitely willing*” or “*somewhat willing*”) or unwilling (“*definitely unwilling*” or “*somewhat unwilling*”). Sociodemographic and military service characteristics were also assessed via self-report.

### Analytic approach

Analyses were conducted using SAS version 9.4 and R Version 4.4.3. All analyses were weighted to account for the complex survey design and to reduce bias and enhance generalizability. Final weights incorporated design weights (i.e., for disproportionate sampling across groups), eligibility adjustment (i.e., for known eligibility of sampled cases), non-response adjustment, and raking (i.e., to ensure alignment with the known veteran population). Non-response analysis indicated response propensity was associated with Veterans Health Administration (VHA) service use, rurality, race/ethnicity, age, and recency of separation [8]. Non-response adjustment accounted for differential nonresponse among sampled cases with known eligibility. As such, findings represent population-based estimates for all living veterans residing in all 50 U.S. states, DC, and Puerto Rico at the time of survey administration (2022). More details on weighting procedures are provided in Supplemental Appendix 1.

For each series of results, unweighted frequencies, weighted percentages, and 95% weighted Wilson confidence intervals (CI) are reported. Domain estimates were performed for weighted results to appropriately estimate variance; additionally, missing data were treated as not missing at completely at random to ensure accurate variance calculation. Group differences (i.e., comparing those who reported access to firearms in their home, those who did not, and those who declined to respond) in weighted frequencies were tested via chi-square tests. Those who had missing data for the firearm access question were excluded from subsequent analyses by including these individuals in their own domain to allow for accurate variance calculation. All weighted prevalence estimates reported met the National Center for Health Statistics Data Presentation Standards for Proportions [32].

## Results

### Population characteristics

Characteristics among veterans overall are included in Supplemental Table 1. Characteristics by household firearm access responses are provided in Supplemental Table 2, and differed by age, sex, gender, sexual orientation, race/ethnicity, region, rurality, branch (Navy and Marines), Army National Guard service, if ever deployed, service era, VHA enrollment, and VHA use.

**Table 1:** Firearm Access, Storage, and Reasons for Having Household Firearms (*n* = 17,396)

	*n*	Weighted *N*	Weighted %	95% CI	
**Access to firearms in the home**					
No	6,461	5,265,208	33.73	32.81	34.66
Yes	9,537	8,961,211	57.40	56.41	58.39
Declined to respond	1,136	1,208,230	7.74	7.16	8.36
Skipped	262	176,672	1.13	0.98	1.31
**Firearms stored locked (i.e.**,** in a gun safe**,** locked cabinet**,** or with a trigger lock)**^1,2^ [missing: *n* = 262]					
None	3,168	2,755,305	31.45	30.25	32.68
Some	2,033	1,950,739	22.27	21.15	23.43
All	4,074	4,053,506	46.28	44.90	47.65
**Firearms stored loaded with ammunition**^1,3^ [missing: *n* = 231]					
All	1,375	1,385,619	15.78	14.80	16.80
Some	2,873	2,915,778	33.20	31.90	34.53
None	5,058	4,480,964	51.02	49.64	52.40
**Reasons for having household firearms** ^1,4^					
Protection against people	6,587	6,637,195	74.27	73.18	75.33
Hunting	4,355	4,029,276	45.09	43.76	46.43
Other sporting use	3,623	3,721,775	41.65	40.29	43.02
A collection	2,621	2,692,743	30.13	28.85	31.45
Protection against animals	2,374	2,353,576	26.34	25.12	27.60
Some other reason^5^	901	879,228	9.84	9.06	10.68

CI = confidence interval

^1^ Among veterans who reported having household firearm access

^2^ 53.72% (95% CI: 52.35–55.10) reported that at least some firearms were stored unlocked (i.e., none or some were stored locked)

^3^ 48.98% (95% CI: 47.60-50.36) reported that at least some firearms (i.e., some or all) were stored loaded

^4^ Respondents could select multiple response options

^5^ Analysis of respondents’ free text responses indicated that “other” reasons included: (a) firearms received as gifts, inherited, or family heirlooms; (b) firearms owned by another family member; (3) their Second Amendment right; (c) proficiency, and (d) the potential for insurrection, rebellion, or other events [uncommonly reported]

### Access to firearms within the home

Over half of veterans (57.40%; 95% CI: 56.41–58.39) indicated that they had access to firearms in their home (Table 1). This percentage (57.40%) does not include the 8.87% (95% CI: 8.28–9.50) whose household firearm access status could not be ascertained: 7.74% (95% CI: 7.16–8.36) selected “decline to respond,” and 1.13% (95% CI: 0.98–1.31) skipped this question.

### Firearm storage

Among veterans with access to household firearm(s), 53.72% (95% CI: 52.35–55.10) reported that at least some were stored unlocked (i.e., none or some stored locked in a gun safe, locked cabinet, or stored with a trigger lock), and 48.98% (95% CI: 47.60–50.36) reported that at least some were stored loaded with ammunition (Table 1).

### Reasons for having household firearms

Among veterans with access to household firearm(s), the most common reason for having household firearms was protection against people (74.27%; 95% CI: 73.18–75.33), followed by hunting (45.09%; 95% CI: 43.76–46.43), other sporting use (41.65%; 95% CI: 40.29–43.02), a collection (30.13%; 95% CI: 28.85–31.45), and protection against animals (26.34%; 95% CI: 25.12–27.60; Table 1). Nearly 10% of veterans (9.84%; 95% CI: 9.06–10.68) described having household firearms for some “other” reason.

### Perceptions of firearms and suicide risk

The majority of veterans (ranging from 55.24% to 71.03%) reported at least some level of agreement with various beliefs about firearm access impacting suicide risk; Table 2). Prevalence significantly differed based on veterans’ responses regarding household firearm access. Specifically, the percentage who endorsed agreement with such beliefs was highest among veterans who indicated that they did not presently have household firearm access (range 71.53% to 84.19%), was lower among those who indicated that they had household firearm access (48.59% to 66.00%), and was lowest among veterans who declined to respond to the household firearm access question (34.38% to 57.67%; Table 3; Supplemental Fig. 1).

**Table 2:** Perceptions of Firearms and Suicide Risk, Willingness to Alter and Discuss Firearm Access, and Prior Firearm Discussions, among Veterans Overall (*n* = 17,134)

	*n*	Weighted *N*	Weighted %	95% CI
**Perceptions of firearms and suicide risk** ^1^				
***Keeping firearms locked and unloaded can reduce risk of suicide*** [missing: *n* = 443]				
Agree	11,841	10,392,702	68.77	67.78, 69.73
Disagree	4,850	4,720,521	31.23	30.27, 32.22
***Temporarily removing firearms from the home can reduce suicide risk*** [missing: *n* = 403]				
Agree	12,050	10,723,197	71.03	70.08, 71.97
Disagree	4,621	4,372,708	28.97	28.03, 29.92
***Having a firearm in the home increases risk of suicide*** [missing: *n* = 463]				
Agree	9,425	8,364,498	55.24	54.21, 56.28
Disagree	7,306	6,776,348	44.76	43.72, 45.79
**Willingness to alter firearm access if thinking about suicide** ^**2**^				
**Unload and/or lock household firearms [missing: n = 1,529]**				
Willing	13,251	12,120,021	84.62	83.83, 85.38
Unwilling	2,354	2,203,022	15.38	14.62, 16.17
**Temporarily remove ammunition for household firearms offsite** [missing: *n* = 1,601]				
Willing	12,108	10,843,942	75.98	75.02, 76.91
Unwilling	3,425	3,429,007	24.02	23.09, 24.98
**Temporarily remove household firearms offsite** [missing: *n* = 1,497]				
Willing	12,016	10,743,627	74.94	73.98, 75.88
Unwilling	3,621	3,592,347	25.06	24.12, 26.02
**Willingness to discuss household firearm access with different individuals** ^**2**^				
**Family member** [missing *n* = 1,025]				
Willing	14,184	13,032,829	88.41	87.70, 89.09
Unwilling	1,925	1,708,165	11.59	10.91, 12.30
**Friend** [missing *n* = 1,082]				
Willing	13,330	12,344,119	83.95	83.15, 84.72
Unwilling	2,722	2,359,958	16.05	15.28, 16.85
**Healthcare provider** [missing *n* = 1,111]				
Willing	11,907	10,616,974	72.42	71.43, 73.39
Unwilling	4,116	4,043,097	27.58	26.61, 28.57
**Prior discussion with healthcare provider(s) about firearm access** [missing *n* = 831]				
No	14,295	12,937,510	87.15	86.38, 87.87
Yes	2,008	1,908,432	12.85	12.13, 13.62

CI = confidence interval. “Overall” included veterans who declined to respond to the question concerning household firearm access

^1^ Agreement was operationalized as endorsement of “a*gree a little*,*” “somewhat agree*,*” “moderately agree*,*”* or “*strongly agree*.” Disagreement was operationalized as endorsement of “*do not agree at all.”*

^2 “^Willing” was operationalized as endorsement of “*somewhat willing*” or “*definitely willing*”; unwilling was operationalized as endorsement of “*somewhat unwilling*” or “*definitely unwilling*.”

**Table 3:** Perceptions of Firearms and Suicide Risk, Willingness to Alter and Discuss Firearm Access, and Prior Firearm Discussions, Stratified based on Responses to the Household Firearm Access Question (*n* = 17,134)

	By Household Firearm Access Response										
	**Yes (n = 9.537)**			**No (n = 6,461)**			**Decline to Respond (n = 1,136)**			Chi-square	*p*-value
	*n*	Weighted %	95% CI	*n*	Weighted %	95% CI	*n*	Weighted %	95% CI		
**Perceptions of firearms and suicide risk** ^**1**^											
***Keeping firearms locked and unloaded can reduce risk of suicide*** [missing: *n* = 443]											
Agree	6,196	64.22	62.90, 65.53	5,019	79.17	77.68, 80.58	626	57.67	53.46, 61.77	214.09	< 0.0001
Disagree	3,217	35.78	34.48, 37.10	1,195	20.83	19.42, 22.32	438	42.33	38.23, 46.54		
***Temporarily removing firearms from the home can reduce suicide risk*** [missing: *n* = 403]											
Agree	6,230	66.00	64.70, 67.28	5,288	84.19	82.86, 85.44	532	51.30	47.12, 55.46	401.37	< 0.0001
Disagree	3,165	34.00	32.72, 35.30	930	15.81	14.56, 17.14	526	48.70	44.54, 52.88		
***Having a firearm in the home increases risk of suicide*** [missing: *n* = 463]											
Agree	4,537	48.59	47.22, 49.96	4,519	71.53	69.96, 73.06	369	34.38	30.64, 38.33	506.36	< 0.0001
Disagree	4,892	51.41	50.04, 52.78	1,714	28.47	26.94, 30.04	700	65.62	61.67, 69.36		
**Willingness to alter firearm access if thinking about suicide** ^**2**^											
**Unload and/or lock household firearms** [missing: *n* = 1,529]											
Willing	7,201	81.31	80.21, 82.36	5,373	92.91	91.93, 93.79	677	73.57	69.51, 77.26	226.66	< 0.0001
Unwilling	1,711	18.69	17.64, 19.79	377	7.09	6.21, 8.07	266	26.43	22.74, 30.49		
**Temporarily remove ammunition for household firearms offsite** [missing: *n* = 1,601]											
Willing	6,314	70.09	68.76, 71.39	5,261	90.90	89.76, 91.91	533	55.49	51.02, 59.87	501.26	< 0.0001
Unwilling	2,554	29.91	28.61, 31.24	469	9.10	8.09, 10.24	402	44.51	40.13, 48.98		
**Temporarily remove household firearms offsite** [missing: *n* = 1,497]											
Willing	6,231	68.72	67.38, 70.02	5,262	90.46	89.32, 91.50	523	54.49	50.03, 58.89	525.52	< 0.0001
Unwilling	2,709	31.28	29.98, 32.62	499	9.54	8.50, 10.68	413	45.51	41.11, 49.97		
**Willingness to discuss household firearm access with different individuals** ^**2**^											
**Family member [missing n = 1,025]**											
Willing	8,105	90.12	89.21, 90.96	5,291	88.48	87.37, 89.50	788	75.46	71.54, 78.99	112.34	< 0.0001
Unwilling	884	9.88	9.04, 10.79	764	11.52	10.50, 12.63	277	24.54	21.01, 28.46		
**Friend [missing n = 1,082]**											
Willing	7,526	85.20	84.18, 86.16	5,103	85.27	83.96, 86.49	701	68.97	65.11, 72.58	115.05	< 0.0001
Unwilling	1,424	14.80	13.84, 15.82	936	14.73	13.51, 16.04	362	31.03	27.42, 34.89		
**Healthcare provider [missing n = 1,111]**											
Willing	6,482	70.75	69.40, 72.06	5,052	83.86	82.58, 85.07	373	34.98	31.14, 39.03	631.87	< 0.0001
Unwilling	2,449	29.25	27.94, 30.60	985	16.14	14.93, 17.42	682	65.02	60.97, 68.86		
**Prior discussion with healthcare provider(s) about firearm access** [missing *n* = 831]											
No	7,953	87.91	86.95, 88.80	5,475	87.77	86.48, 88.96	867	78.81	74.98, 82.19	37.82	< 0.0001
Yes	1,060	12.09	11.20, 13.05	731	12.23	11.04, 13.52	217	21.19	17.81, 25.02		

CI = confidence interval

^1^ Agreement was operationalized as endorsement of “a*gree a little*,*” “somewhat agree*,*” “moderately agree*,*”* or “*strongly agree*.” Disagreement was operationalized as endorsement of “*do not agree at all.”* firearm access question

^2 “^Willing” was operationalized as endorsement of “*somewhat willing*” or “*definitely willing*”; unwilling was operationalized as endorsement of “*somewhat unwilling*” or “*definitely unwilling*.”

### Willingness to alter firearm access if thinking about suicide

Among veterans with access to household firearm(s), the majority indicated at least some willingness to alter their firearm access if they were thinking about suicide (Table 2), including to unload and/or lock firearms (84.62%; 95% CI: 83.83–85.38), temporarily remove ammunition for household firearms offsite (75.98%; 95% CI: 75.02–76.91), or temporarily remove household firearms offsite (74.94%; 95% CI: 73.98–75.88), if experiencing thoughts of suicide. These percentages significantly differed based on veterans’ responses regarding household firearm access: the percentage who endorsed being willing to engage in each of these behaviors was highest among veterans who did not have household firearm access (90.46% to 92.91%), was lower among veterans who indicated that they had household firearm access (68.72% to 81.31%), and was the lowest among veterans who declined to respond to the household firearm access question (54.49% to 73.57%; Table 3; Supplemental Fig. 1).

### Firearm discussions

#### Willingness to discuss

Overall, the majority of veterans expressed being willing to discuss household firearms with various individuals, with the highest percentage endorsing willingness to discuss firearms with a family member (88.41%; 95% CI:87.70–89.09), followed by a friend (83.95%; 95% CI:83.15–84.72), and the lowest percentage with a healthcare provider (72.42%; 95% CI:71.43–73.39) (Table 2). Again, however, the percentages willing to discuss household firearms significantly differed based on veterans’ responses regarding household firearm access (Table 3; Supplemental Fig. 1). Specifically, higher proportions of veterans who indicated that they had (90.12%; 95% CI: 89.21–90.96) or did not have (88.48%; 95% CI:87.37–89.50) household firearm access, relative to veterans who declined to respond to the household firearm question (75.46%; 95% CI: 71.54–78.99), indicated that they would be willing to discuss household firearms with a family member. Similar results were observed regarding willingness to discuss household firearms with a friend, with higher proportions of veterans who had (85.20%; 95% CI: 84.18–86.16) or did not have (85.27%; 95% CI: 83.96–86.49) household firearm access being willing to do so, relative to veterans who declined to respond to the household firearm question (68.97%; 95% CI: 65.11–72.58). Lastly, the proportion of veterans willing to discuss household firearms with a healthcare provider was highest among veterans who reported not presently having household firearms (83.86%; 95% CI: 82.58–85.07), was lower among veterans who reported having household firearms (70.75%; 95% CI: 69.40–72.06), and was, by far, the lowest among veterans who declined to respond to the household firearm question (34.98%; 95% CI: 31.14–39.03).

#### Prior discussions

Only a minority of veterans (12.85%; 95% CI: 12.13–13.62) indicated that any healthcare provider had ever asked them about their access to firearms (Table 2). The proportions of veterans reporting this also differed significantly by response to the household firearm access question, with higher proportions of veterans who declined to answer the household firearm access question (21.19%; 95% CI: 17.81–25.02) reporting that any healthcare provider had ever asked them about their access to firearms, relative to veterans with (12.09%; 95% CI: 11.20–13.05) and without household firearms (12.23%; 95% CI: 11.04–13.52) (Table 3; Supplemental Fig. 1).

## Discussion

Access to firearms is associated with suicide [3, 19]. As such, it is vital to better understand veterans’ access to firearms, their storage behaviors, and their willingness to engage in different behaviors to reduce risk for firearm injury and firearm suicide when suicide risk is elevated. This study builds upon prior research that has been instrumental to understanding population-based rates of firearm ownership and storage among U.S. veterans [14–17], providing recent (2022) population-based estimates of U.S. veterans’ firearm access, storage practices, firearm-related perceptions, and willingness to engage in firearm discussions and behaviors when thinking about suicide. In doing so, this study provides a more comprehensive portrait of factors which may impact veterans’ risk for firearm injury and suicide.

### Household firearm access

Our findings indicate that, in 2022, over half of veterans (57.40%; 95% CI: 56.41–58.39)—corresponding to approximately 8.96 million veterans—had access to firearms in their home. Importantly, this figure may be an underestimate, as 8.87% of respondents declined to respond to or skipped the access question. Although prior studies have focused on personal or household firearm ownership, the broader construct of access may be more relevant for suicide prevention. While measurement differences across studies limit direct comparison, our access estimate exceeds prior estimates of personal ownership among veterans, including from the 2015 NFS (44.9% [95% CI: 41.3–48.6 [14]) and the 2022 NHRVS (50.9% [95% CI: 48.0–53.9 [15]). These differences cannot be interpreted as evidence of increasing ownership. However, national context may help to situate these findings. Multiple data sources—including FBI National Instant Criminal Background Check System (NICS) statistics—indicate that background checks increased substantially during the COVID-19 pandemic, with 2020 and 2021 among the highest years on record for firearm purchase–related checks nationwide [33]. These U.S. level patterns suggest broader increases in firearm acquisition during the pandemic period. Importantly, because our measure captured household access and prior veteran estimates reflected personal ownership, these sources are not directly comparable. Accordingly, our findings should not be interpreted as indicating increased firearm ownership among veterans specifically, but rather as underscoring the importance of continued monitoring of firearm access in this population.

### Firearm storage

Additionally, in 2022, over half of veterans with household firearm access stored some or all household firearms unlocked (53.72%; 95% CI: 52.35–55.10). This estimate is *lower* than the 2015 NFS estimate of 66% (95% CI: 60.4–70.3) among veteran firearm owners [16]. We also found that nearly half of veterans with household firearm access (48.98%; 95% CI: 47.60–50.36) reported storing some or all household firearms loaded with ammunition. This is comparable to the 2015 NFS (46.7% [95% CI: 41.5–51.9] [16]; yet *higher* than the 2022 NHRVS estimate (39.9% [95% CI: 37.0–42.8] [17]), among veteran firearm owners. Differences across studies may reflect methodological variation in measuring access versus personal or household ownership, as well as actual shifts in storage practices over time. In addition to ongoing VA initiatives promoting secure storage (e.g., distribution of free cable locks [5]), broader temporal changes in firearm storage behaviors may also contribute. For example, Conrick and colleagues observed increases in secure storage among both veterans and nonveterans living in households with firearms in Washington State between 2013 and 2022 [34]. Although this evidence is from a single state and may not reflect national trends, it suggests the possibility of wider changes that warrant further investigation. Future research across longer periods is necessary to determine whether veterans’ storage behaviors are changing nationally and to identify potential drivers of such changes. Regardless, our findings suggest that, in 2022, many veterans resided in homes with at least one firearm stored unlocked (approximately 4.71 million) and/or loaded (approximately 4.30 million).

### Veterans’ willingness to alter firearm access and risk perceptions

Perhaps the most striking finding from the present study is the extent to which veterans reported being willing to engage in specific behaviors to reduce their firearm-related suicide risk, such as unloading and/or locking household firearms (84.62%; 95% CI: 83.83–85.38) or temporarily removing ammunition (75.98%; 95% CI: 75.02–76.91) or household firearms (74.94%; 95% CI: 73.98–75.88) offsite, if they were thinking about suicide. These percentages were lower among veterans with household firearm access (ranging from 68.72% to 81.31%) and among veterans who declined to respond to the question about firearm access (ranging from 54.49% to 73.57%), yet still endorsed by the majority of these veterans. As such, the current findings lend important support for clinical and public health efforts aimed at promoting secure firearm storage (both in- and out-of-home) when veterans are at elevated risk for suicide.

Moreover, the high percentage of veterans who indicated that they would be willing to engage in different behaviors to reduce their firearm access if thinking about suicide may relate to veterans’ firearm perceptions: the majority of veterans agreed that securely storing firearms (i.e., locked and unloaded; 68.77%; 95% CI: 67.78–69.73) and temporarily removing firearms from the home (71.03%; 95% CI: 70.08–71.97) can reduce suicide risk. A lower percentage of veterans (55.24%; 95% CI: 54.21–56.28) agreed that having a firearm in the home increases risk of suicide; however, this percentage (even when focusing only on veterans with household firearm access, in which 48.59% [95% CI: 47.22–49.96] endorsed some level of agreement, or veterans who declined to respond, in which 34.38% [95% CI: 30.64–38.33] endorsed some level of agreement) is higher than the 2015 NFS estimate, in which only 6.3% of veteran firearm owners agreed or strongly agreed that the presence of a firearm within the home increases risk for suicide among household members [23]. An increase over time in the extent to which veterans agree with statements regarding household firearms increasing suicide risk for oneself or other household members could, in part, relate to increased public health messaging [4–6]; however, even if messaging campaigns have been effective, it is unlikely that any single factor could explain such a major difference. Given the different focus (oneself in ASCEND vs. other household members in the NFS) and response options in each study,^1^ concluding that there have been true changes in veterans’ firearm risk perceptions over time is not possible, and future research is warranted to elucidate such trends.

### Firearm discussions

Another promising finding was the high percentage of veterans who indicated that they would be willing to discuss household firearms with others (72.42% to 88.41% among veterans overall, depending on the discussant). The percentage of veterans willing to discuss household firearms was highest for family members (88.41%; 95% CI:87.70–89.09), followed by a friend (83.95%; 95% CI:83.15–84.72). This is consistent with prior qualitative studies (e.g. [25]), and underscores the importance of ensuring that veterans’ family members are well-equipped to have firearm discussions and be included in firearm LMS efforts, if appropriate, which may entail providing them with basic resources and education [35, 36]. Initiatives to begin to address this are underway [36, 37], and continued research remains important to understand optimal ways to include veterans’ family members and other trusted sources (e.g., friends) in firearm LMS, particularly given some of the complexities of doing so [38].

Moreover, while the percentage of veterans willing to discuss household firearms was lowest when pertaining to discussions with healthcare providers, it was still quite high (72.42%; 95% CI: 71.43–73.39; 70.75% [95% CI: 69.40–72.06] among those with firearm access), aligning with prior research. For example, in 2019, 79.4% (95% CI, 75.5%–82.8%) of veteran firearm owners indicated that healthcare providers should^2^ discuss firearms and firearm safety when a patient or family member is at risk for suicide [38]. Indeed, qualitative research suggests that veterans generally believe that discussing firearm safety is acceptable, even if doing so can be uncomfortable [27]. Notably, in addition to the 72.42% who indicated they would be somewhat or definitely willing to discuss firearms with a primary care or mental health provider, another 7.54% (95% CI: 6.95–8.16; 8.42% [95% CI: 7.57–9.35] among those with firearm access) indicated they were ‘somewhat unwilling.’ Although we characterized these respondents as ‘unwilling’ in our analyses, this response may indicate some potential for clinician-led counseling. Given prior work elucidating the variety of patient concerns that impact the acceptability of these conversations, such respondents may be indicating cautious endorsement of the role of clinicians in providing LMS counseling. Additional work is needed to improve understanding of the best approaches to address firearm-specific suicide risk among the 20.04% (95% CI: 19.18, 20.94) of veterans who report being ‘definitely unwilling’ to discuss firearms with a provider.

Only 12.85% (95% CI: 12.13–13.62)^3^ of veterans reported that a healthcare provider had ever asked them about their access to firearms. This is relatively similar to 2019 findings with veteran firearm owners, in which only 9.2% (95% CI 6.8–12.3) reported that a healthcare provider had ever spoken with them about firearm safety [24]. Provider concerns about discussing firearms in healthcare settings may be one potential reason for this [39, 40], despite research suggesting that veterans are open to discussing firearms. Although we did not examine the extent to which veterans at elevated risk for suicide reported having a prior discussion about firearms with a healthcare provider, prior research suggests that 32% of veterans have experienced lifetime suicidal ideation; [8]). Thus, our findings suggest that there is likely a need to increase understanding of patient-, clinician-, and system-level barriers to firearm-related discussions, particularly among veterans at increased risk for suicide. Given various VA healthcare provider trainings and initiatives implemented surrounding firearm LMS since ASCEND data were collected in 2022, future research is warranted to understand if healthcare providers’ firearm discussions with veterans have increased over time, including in specific healthcare settings [24].

### Declining to respond

Lastly, a large portion of veterans (7.74%; 95% CI: 7.16–8.36) selected “decline to respond” to the household firearm access question, which is comparable to prior surveys (e.g., 6.8%; [41]; 11.8% [42]). Our findings suggest that individuals who decline to respond to firearm access questions may comprise a distinct group with respect to their firearm risk perceptions and willingness to engage in different firearm-related behaviors. Veterans who declined to respond reported the lowest agreement with statements about firearm access and storage impacting suicide risk (34.38%-57.67%), the lowest willingness to change their firearm access if they were thinking about suicide (54.49%-73.57% willing), and the lowest willingness to discuss firearms with others (68.97% and 75.46% willing for friends and family, respectively), particularly with healthcare providers (34.98%; 95% CI: 31.14–39.03). Conversely, they comprised the highest percentage who reported having had a prior firearm-related discussion with a healthcare provider (21.19%; 95% CI: 17.81–25.02). This pattern could reflect negative prior experiences with such discussions or increased recall if the conversation was perceived as uncomfortable or inappropriate. These findings may also indicate a need to improve the delivery of firearm-related conversations with some veterans, particularly if negative prior experiences are contributing to reluctance to engage in future discussions. Although additional research is needed to better understand the reasons underlying and driving this reluctance, these patterns suggest that veterans who decline to respond to firearm access questions may be less amenable to firearm lethal means safety discussions and that alternative strategies for reducing their firearm suicide risk may be warranted.

Prior research has begun to explore characteristics of veterans who decline to respond to firearm questions. In a study of military personnel and veterans in a primary care setting, there were relatively few demographic or clinical differences reported between those who declined to respond (who were, however, more likely to screen positive for depression and endorse experiencing recent thoughts of death or self-harm) and those who reported having a firearm in or around the home, leading the authors to conclude that those who declined were relatively similar to those who endorsed having a firearm [41]. Other studies have also explored the characteristics of those who declined to respond to a firearm question in the general U.S. adult population and identified some differences (e.g. [42]). Continued research to better understand veterans who decline to respond to firearm access questions and identify ways to overcome barriers to obtaining valid and reliable responses from them in research is warranted.

### Limitations

These findings should be interpreted in the context of specific limitations. While firearm access is a key risk factor for suicide, it represents only one component of a broader constellation of influences on suicide risk. The goal of this analysis was not to identify the full range of factors contributing to firearm suicide risk among veterans, and our findings should be interpreted as characterizing patterns of firearm access and related behaviors rather than explaining suicide outcomes. Other influences—such as mental health conditions, economic strain, and social or geographic isolation—also play important roles in shaping suicide vulnerability across populations [43–46]. Additionally, although we asked about access to household firearms, we did not ask about personal firearm ownership, which precludes comparison to other studies, such as the 2015 NFS. In addition, item-level nonresponse, although low, may have introduced bias if respondents who skipped firearm-related questions differed from those who answered. While our analytic approach incorporated missing data into variance estimation, some residual uncertainty due to question-level nonresponse may remain. Furthermore, a substantial percentage of veterans declined to respond to the question about household firearm access, which likely resulted in an underestimate of household firearm access in the veteran population. We also asked about veterans’ willingness to engage in various behaviors if they were thinking about suicide and their willingness to discuss firearms with different individuals; yet social desirability bias is important to consider, and the extent to individuals’ responses map onto actual behavior in such circumstances is not known. In addition, as discussed, we included “somewhat unwilling” in the “unwilling” category; however, it is possible that these respondents (who did not select “definitely unwilling”) have a low level of willingness to engage in these behaviors or discussions. Recall bias also could have impacted veterans’ responses regarding prior firearm discussions with healthcare providers; future research using alternate methods (e.g., analysis of medical records) could help to augment self-report regarding the extent to which these conversations occur in different healthcare settings. Lastly, as we did not examine subgroup differences in this analysis, future research focused on these differences will be important. Given elevated suicide risk among older veterans, examining age-related variation in firearm access, storage practices, and related perceptions represents an important direction for future work [1].

## Conclusions

Given the strong association between population-level firearm access prevalence and suicide, these findings can inform firearm suicide prevention efforts for U.S. veterans. Our findings suggest that, in 2022, access to firearms within the home was prevalent among veterans, with many storing their firearms in a manner (i.e., loaded, unlocked) that may heighten their risk for firearm injury and suicide. Results also suggest willingness among veterans to include family members (and others) in firearm discussions, along with a need to increase provider firearm LMS discussions, which may facilitate preventing firearm injury and suicide among veterans and other household members. Additionally, veterans who decline to respond to firearm access questions may comprise a distinct group with respect to their firearm risk perceptions and willingness to engage in firearm discussions and firearm-related behaviors aimed at decreasing their firearm suicide risk; future research to better understand and characterize these veterans is essential. Finally, continued research is necessary to examine how veterans’ firearm storage, risk perceptions, preferences, and discussions change over time.

## Supplementary Information

Supplementary Material 1: Provides methodological details on response rate calculation and weighting procedures; firearm-related survey content; and descriptive characteristics of veterans overall and by responses to the household firearm access question. Also includes visual displays comparing firearm-related responses, stratified by household firearm access responses.

## Data Availability

Data utilized in this study cannot be publicly shared because of privacy and confidentiality requirements. Data are available from the VA R&D Committee (phone contact: 303-399-8020) for researchers who meet the criteria for access to confidential study data.

## Footnotes

1 ASCEND conceptualized agreement as responses of “*agree a little*,” “*somewhat agree*,” “*moderately agree*,” or “*strongly agree*,” whereas lack of agreement was conceptualized as “*do not agree at all*.” In the 2015-NFS, the response options other than “*agree”* or “*strongly agree*” included “*neither*,” “*disagree*,” or “*strongly agree*” [26].

2 Sometimes or always.

3 12.09% (95% CI: 11.20–13.05) among Veterans who reported household firearm access.

## References

[1] Department of Veterans Affairs. National Veteran Suicide Prevention Annual Report: Part 2 of 2: Report Findings [Internet]. 2025. Available from: https://www.mentalhealth.va.gov/docs/data-sheets/2025/2025_National_Veteran_Suicide_Prevention_Annual_Report_PART_2_FINAL.pdf.

[2] Conner A, Azrael D, Miller M. Suicide case-fatality rates in the United States, 2007 to 2014: A nationwide population-based study. Ann Intern Med. 2019;171(12):885–95. 10.7326/M19-1324 31791066

[3] Anglemyer A, Horvath T, Rutherford G. The accessibility of firearms and risk for suicide and homicide victimization among household members: A systematic review and meta-analysis. Ann Intern Med. 2014;160(2):2. 10.7326/M13-1301 24592495

[4] Department of Veterans Affairs. National Strategy of Prevention Veteran Suicide 2018–2028 [Internet]. 2018. Available from: https://www.mentalhealth.va.gov/suicide_prevention/docs/Office-of-Mental-Health-and-Suicide-Prevention-National-Strategy-for-Preventing-Veterans-Suicide.pdf.

[5] Department of Veterans Affairs. Keep it Secure [Internet]. Available from: https://www.va.gov/reach/lethal-means/.

[6] Department of Veterans Affairs. National Veteran Suicide Prevention Annual Report: Part 2 of 2 [Internet]. 2024. Available from: https://www.mentalhealth.va.gov/docs/data-sheets/2024/2024-Annual-Report-Part-2-of-2_508.pdf.

[7] Hoffmire CA, Mohatt NV, Holliday R, Barnes SM, Brenner LA, Monteith LL. ASCEND for Veteran Suicide Prevention: Enhancing surveillance to save lives. Psychiatry Res. 2022;310(114432). 10.1016/j.psychres.2022.114432. 10.1016/j.psychres.2022.114432 35150969

[8] Hoffmire CA, Barnes SM, Holliday R, Kittel JA, Schneider AL, Brenner LA, S Veterans. Non-fatal suicidal self-directed violence among U. S. Veterans (2022): The Assessing Social and Community Environments with National Data (ASCEND) for Veteran Suicide Prevention Study. Am J Epidemiol. 2024. 10.1093/aje/kwae461. 10.1093/aje/kwae461 PMC13343375 39694860

[9] Wendleton L, Hoffmire CA, Drop S, Flower M, Jones R, Nolan JP, et al. Veteran engagement in survey research to prevent suicide. Progress Community Health Partnerships: Res Educ Action. 2023;17(1):129–34. 10.1353/cpr.2023.0017. 10.1353/cpr.2023.0017 37462581

[10] Miller M, Zhang W, Azrael D. Firearm purchasing during the COVID-19 pandemic: Results from the 2021 National Firearms Survey. Ann Inter Med. 2022;175(2):219–25. 10.7326/M21-3423 PMC8697522 34928699

[11] Wu TY, Hsieh HF, Chow CM, Yang X, Resnicow K, Zimmerman M. Examining racism and firearm-related risks among Asian Americans in the United States during the COVID-19 pandemic. Prev Med Rep. 2022;27:101800. 35656206 10.1016/j.pmedr.2022.101800 PMC9152798

[12] Monteith LL, Miller CN, Polzer E, Holliday R, Hoffmire CA, Iglesias CA, et al. Feel the need to prepare for Armageddon even though I do not believe it will happen: Women Veterans Firearm Beliefs and Behaviors during the COVID-19 Pandemic, Associations with Military Sexual Assault and Posttraumatic Stress Disorder Symptoms. PLoS ONE. 2023;10(2):e0280431. 10.1371/journal.pone.0280431 PMC9917279 36763646

[13] Valek R, Ward JA, Jones V, Crifasi CK. Political violence, racial violence, and new gun ownership: Results from the 2023 National Survey of Gun Policy. Injury Epidemiol. 2024;6(1):48. 10.1186/s40621-024-00527-z PMC11378614 39243093

[14] Cleveland EC, Azrael D, Simonetti JA, Miller M. Firearm ownership among American veterans: Findings from the 2015 National Firearm Survey. Injury Epidemiol. 2017;4(1):1. 10.1186/s40621-017-0130-y PMC5735043 29256160

[15] Fischer IC, Aunon FM, Nichter B, Hill ML, Panza KE, Kline AC, et al. Firearm ownership among a nationally representative sample of US veterans. Am J Prev Med. 2023;1(6):1129–33. 10.1016/j.amepre.2023.06.013 37354925

[16] Simonetti JA, Azrael D, Rowhani-Rahbar A, Miller M. Firearm storage practices among American veterans. Am J Prev Med. 2018;1(4):445–54. 10.1016/j.amepre.2018.04.014 30166080

[17] Nichter B, Hill ML, Fischer I, Panza KE, Kline AC, Na PJ et al. Firearm storage practices among military veterans in the United States: Findings from a nationally representative survey. J Affect Disord, 2024;351:82–9. 10.1016/j.jad.2024.01.179 38280567

[18] Miller M, Wertz J, Swanson SA, Simonetti JA, Zhang Y, Azrael DR. Firearm storage and firearm suicide. JAMA Netw Open. 2025;1(7):e2519266. 10.1001/jamanetworkopen.2025.19266 PMC12235496 40622712

[19] Miller M, Swanson SA, Azrael D. Are we missing something pertinent? A bias analysis of unmeasured confounding in the firearm-suicide literature. Epidemiol Rev. 2016;1(1):62–9. 10.1093/epirev/mxv011 26769723

[20] Miller M, Zhang Y, Prince L, Swanson SA, Wintemute GJ, Holsinger EE, et al. Suicide deaths among women in California living with handgun owners vs those living with other adults in handgun-free homes, 2004–2016. JAMA psychiatry. 2022;1(6):582–8. 10.1001/jamapsychiatry.2022.0793 PMC9047728 35476016

[21] Monteith LL, Holliday R, Miller CN, Schneider AL, Brenner LA, Hoffmire CA. Prevalence and correlates of firearm access among post-9/11 US women veterans using reproductive healthcare: A cross-sectional survey. J Gen Intern Med. Sep; 2022;37(Suppl 3):714–23. 36042091 10.1007/s11606-022-07587-1 PMC9481791

[22] Polzer ER, Holiday R, Rohs CM, Thomas SM, Miller CN, Simonetti JA et al. Women veterans’ perspectives, experiences, and preferences for firearm lethal means counseling discussions. PLOS ONE. 2023;18(12). 10.1371/journal.pone.0295042 PMC10699600 38055694

[23] Simonetti JA, Azrael D, Miller M. Firearm storage practices and risk perceptions among a nationally representative sample of US veterans with and without self-harm risk factors. Suicide Life‐Threatening Behav. 2019;49(3):3. 10.1111/sltb.12463 29658142

[24] Simonetti JA, Azrael D, Zhang W, Miller M. Receipt of clinician-delivered firearm safety counseling among US Veterans: Results from a 2019 national survey. Suicide Life‐Threatening Behav. Dec; 2022;52(6):1121–5. 10.1111/sltb.12906 35924483

[25] Simonetti JA, Dorsey Holliman B, Holiday R, Brenner LA, Monteith LL. Firearm-related experiences and perceptions among United States male veterans: A qualitative interview study. PLoS ONE. 2020;15(3):0230135. 10.1371/journal.pone.0230135 PMC7064196 32155211

[26] Dobscha SK, Clark KD, Newell S, Kenyon EA, Karras E, Simonetti JA, et al. Strategies for discussing firearms storage safety in primary care: Veteran perspectives. J Gen Intern Med. 2021;36(6):6. 10.1007/s11606-020-06412-x. 10.1007/s11606-020-06412-x PMC8175613 33501537

[27] Newell S, Kenyon E, Clark KD, Elliott V, Rynerson A, Gerrity MS, et al. Veterans are agreeable to discussions about firearms safety in primary care. J Am Board Fam Med. 2021;34(2):338. 10.3122/jabfm.2021.02.200261. 33833002 10.3122/jabfm.2021.02.200261

[28] Lafferty M, O’Neill A, Cerra N, Maxim L, Mulcahy A, Wyse JJ, et al. Let’s talk about firearms: Perspectives of older veterans and VA clinicians on universal and dementia-specific firearm safety discussions. Clin gerontologist. 2024;7(4):544–54. 10.1080/07317115.2023.2254292 37665611

[29] Monteith LL, Kittel JA, Holliday R, Schneider AL, Herring-Nathan E, Krishnamurti LS, et al. Suicidal ideation and non-fatal suicidal self-directed violence prevalence and associations among Veterans residing in U.S. Pacific Island Territories: Guam, American Samoa, and the Northern Mariana Islands. PLoS ONE. 2025;20(6):0326533. 10.1371/journal.pone.0326533. 10.1371/journal.pone.0326533 PMC12176182 40531952

[30] Hoffmire CA, Kittel JA, Holliday R, Morano TT, Imai Y, Monteith LL. Gender differences in non-fatal suicide self-directed violence prevalence and methods among US v eterans. J Psychiatr Res. 2025;191:653–63. 10.1016/j.jpsychires.2025.10.013 41082831

[31] American Association for Public Opinion Research. Standard definitions: Final dispositions of case codes and outcome rates for surveys [Internet]. 10th edn. American Association for Public Opinion Research. 2023. Available from: https://aapor.org/wp-content/uploads/2024/03/Standards-Definitions-10th-edition.pdf.

[32] Parker JD, Talih M, Malec DJ, Beresovsky V, Carroll M, Gonzalez JF, et al. Data Presentation Standards for Proportions. PubMed PMID: National Center for Health Statistics; 2017. p. 30248016. 30248016

[33] U.S. Department of Justice, Federal Bureau of Investigation, Criminal Justice Information Services Division. National Instant Criminal Background Check System Operational Report 2020–2021 [Internet]. 2022. Available from: https://www.fbi.gov/file-repository/cjis/nics-2020-2021-operations-report.pdf.

[34] Conrick KM, Banks S, Schleimer JP, Gomez A, Pallickaparambil AJ, Ta M, et al. Household firearm storage practices. JAMA Netw Open. 2025;8(7):e2518960. 40608340 10.1001/jamanetworkopen.2025.18960 PMC12232188

[35] Monteith LL, Polzer ER, Rohs CM, Thomas SM, Holliday R, Miller CN, et al. Spouses have a huge role in preventing firearm suicide among women veterans: Partners’ perspectives, experiences, and needs. Women’s Stud Int Forum. 2024;1:105:102920.

[36] DeBeer BB, Matthieu MM, Degutis LC, Clafferty S, Blessing A, Quan C, et al. Firearms lethal means safety among veterans: Attitudes toward involving a concerned significant other. J Military Veteran Family Health. 2025;1(1):23–31. 10.3138/jmvfh-2023-0094 PMC13293169 42533921

[37] Sayers S, Brown LA, Swinkels C, May A, Daniel SS, Crasta D et al. A family member-focused lethal means safety intervention for the prevention of veteran suicide: A clinical pilot study. Cogn Behav Pract. 2025;33(2):330–41.

[38] Aunon FM, Azrael D, Simonetti JA, Miller M. Beliefs among veteran firearm owners regarding whether clinicians should discuss firearm safety with patients. JAMA Netw open. 2023;1(6):e2321219. 10.1001/jamanetworkopen.2023.21219 PMC10311384 37382951

[39] Roszko PJ, Ameli J, Carter PM, Cunningham RM, Ranney ML. Clinician attitudes, screening practices, and interventions to reduce firearm-related injury. Epidemiol Rev. 2016;1(1):87–110. 10.1093/epirev/mxv005 PMC7297261 26905894

[40] Clark KD, Newell S, Kenyon EA, Karras E, Simonetti JA, Gerrity M, et al. Firearms storage safety discussions in VA primary care: Staff perspectives. Gen Hosp Psychiatry. 2021;72:96–101. 34416678 10.1016/j.genhosppsych.2021.07.007

[41] Bryan CJ, Bryan AO, May AM, Harris JA, Baker JC. Depression, suicide risk, and declining to answer firearm-related survey items among military personnel and veterans. Suicide Life‐Threatening Behav. 2021;51(2):197–202. 10.1111/sltb.12694 33876490

[42] Daruwala SE, Bauder CR, Bozzay ML, Bryan CJ. Nonresponse to an item assessing firearm ownership: Associations with suicide risk and emotional distress. Suicide Life-Threatening Behav. Feb; 2025;55(1):e13121. 10.1111/sltb.13121 PMC11687411 39210721

[43] Denneson LM, Bollinger MJ, Phillips R, Chen JI, Carlson KF. County characteristics and veteran suicide in the United States, 2011–2018. Am J Prev Med. 2024;67(5):689–97. 38906428 10.1016/j.amepre.2024.06.011

[44] Mertz-Bynum NE, Bilicki DJ, Brodeur KR, Hirko KA. Rural-urban differences in suicide and firearm-r elated suicide deaths across geographic regions in the United States. Am J Prev Med. 2026;108394. 10.1016/j.amepre.2026.108394 42031032

[45] Schafer KM, Duffy M, Kennedy G, Stentz L, Leon J, Herrerias G, et al. Suicidal ideation, suicide attempts, and suicide death among veterans and service members: A comprehensive meta-analysis of risk factors. Military Psychol. 2022;34(2):129–46. 10.1080/08995605.2021.1976544 PMC10013359 38536290

[46] Richardson T, Elliott P, Roberts R. The relationship between personal unsecured debt and mental and physical health: A systematic review and meta-analysis. Clin Psychol Rev. 2013;33(8):1148–62. 24121465 10.1016/j.cpr.2013.08.009

