# Supplementary material for: Household firearm access, storage, perceptions, and discussions among U.S. military veterans: population-based estimates from the 2022 ASCEND survey
Source: Inj Epidemiol. 2026 May 31;13:54. doi: 10.1186/s40621-026-00692-3 (PMC13436288; doi:10.1186/s40621-026-00692-3)
Supplement: Supplementary file 1 [file 40621_2026_692_MOESM1_ESM.pdf]

Supplemental Materials

Content	Description	Page number(s)
Supplemental Appendix 1	Response Rate Calculation and Weighting Details	2
Supplemental Appendix 2	ASCEND Wave 1 Firearm Questions and Scoring	3-5
Supplemental Table 1	Characteristics of Veterans Overall (n=17,396)	6-7
Supplemental Table 2	Characteristics, by Veterans' Responses to the Household Firearm Access Response (n=17,134)	8-10
Supplemental Figure 1	Visual Displays Comparing Perceptions of Firearms and Suicide Risk, Willingness to Alter and Discuss Firearm Access, and Prior Firearms Discussions, Stratified based on Responses to the Household Firearm Access Question	11-12

Supplemental Appendix 1: Response Rate Calculation and Weighting Details

Response Rate Calculation

The response rate was calculated using the American Association for Public Opinion Research (AAPOR) standard definition for Response Rate 3 (RR3). RR3 is the most commonly reported response rate in survey research and is often regarded as the "gold standard." This metric uses a specific estimation procedure to account for sampled individuals whose eligibility cannot be directly determined, by estimating the proportion of cases with unknown eligibility that are likely to be eligible (AAPOR, 2023).

Weighting

The survey sample was selected from the full sampling frame of living U.S. veterans as of June 2021. Specific subgroups (i.e., women veterans, recently separated veterans, veterans of color) were oversampled to allow for subgroup analyses. To achieve this, stratified sampling occurred across 312 strata created from a cross-tabulation of race/ethnicity, state, sex, and separation status. To account for this sampling design, as well as to account for eligibility and non-response bias, survey weights were constructed for use in analyses. The following weighting steps were performed:

- Design weights: These weights accounted for oversampling. The design weight was calculated as the inverse of the probability of selection.
- Eligibility adjustment: This adjustment incorporated the known eligibility status of sampled cases into the weighting process.
- Non-response adjustment: Analysis of veterans who did not respond indicated that response propensity was associated with age, race/ethnicity, recency of separation, rurality, and recent use of Veterans Health Administration (VHA) care. Younger and recently separated veterans, veterans of color, veterans residing in rural areas, and veterans who had not recently used VHA care were less likely to respond. Thus, we applied a non-response adjustment to the eligibility-adjusted weights to account for differential non-response among sampled cases with known eligibility.
- Raking: We raked the non-response–adjusted weights to align the final survey weights with benchmarks from the known veteran population (e.g., ensuring that the weighted age and sex distributions matched those of the original sampling frame, accounting for eligibility). This procedure also helped address residual noncoverage and nonresponse.

The overall design effect for the ASCEND Wave 1 main sample was estimated to be 1.53.

Final raked weights were used in all analyses via the appropriate procedures. Domain analysis was conducted, and missing data were treated as not missing completely at random to ensure the most accurate variance estimation. Additionally, Wilson confidence intervals were calculated for weighted proportions as these intervals provide better coverage for large samples (Oliver & May, 2006).

References

American Association Public Opinion Research. Standard Definitions: Final Dispositions of Case Codes and Outcome Rates for Surveys [Internet]. 9th ed. American Association for Public Opinion Research; 2023. Available from: https://www.aapor.org/Standards-Ethics/Standard-Definitions-(1).aspx.

Olivier J, May WL. Weighted confidence interval construction for binomial parameters. Stat Methods Med Res. 2006 Feb;15(1):37-46. doi: 10.1191/0962280206sm425oa. PMID: 16477947.

Supplemental Appendix 2: ASCEND Wave 1 Firearm Questions and Scoring

SECTION H. FIREARM ACCESS

The next questions are about working firearms (i.e., firearms capable of being fired). We use the word "firearm" to refer to any pistols, revolvers, shotguns, and rifles. For the purposes of these questions, the term firearm does not include air guns, bb guns, starter pistols, or paintball guns.

We understand that there are many reasons for owning and storing firearms. We are asking these questions to better understand Veterans' firearm-related behaviors and preferences. These questions may be particularly sensitive for some people. As a reminder, you do not have to answer any question you do not wish to answer. Your participation is voluntary. We just ask that you answer to the best of your ability.

Information you provide here is confidential, will not be shared outside of the research team, and will only be used for research purposes. Individual responses will not be shared outside of the research team, and your responses to these items will not affect any of your rights, privileges, or benefits in any way.

Access to firearms within the home

- Asked of all respondents, with the option to decline to respond

53 Do you have access to firearms in your home?

1☐ Yes ➡ *[GO TO Q53B]*
2☐ No
99☐ Decline to respond ➡ *[GO TO Q56]*

Reasons for having household firearms

- Asked only of respondents who endorsed having access to firearms in their home
- Respondents could endorse multiple response options
- "Other" responses were examined to provide cursory information regarding additional reasons for having household firearms

53b For what reasons do you have household firearms?
Select all that apply.

1☐ For protection against people
2☐ For protection against animals
3☐ For hunting
4☐ For other sporting use
5☐ For a collection
6☐ For some other reason, *please specify:*

Firearms stored locked

- Asked only of respondents who endorsed having access to firearms in their home

54 To what extent are the firearm(s) in your home stored in a gun safe, locked cabinet, or stored with a trigger lock?

1☐ All are stored in a gun safe, locked cabinet, or with a trigger lock
2☐ Some are stored in a gun safe, locked cabinet, or with a trigger lock
3☐ None are stored in a gun safe, locked cabinet, or with a trigger lock

Firearms stored loaded

- Asked only of respondents who endorsed having access to firearms in their home

55 To what extent are the firearms in your home stored loaded with ammunition?

3☐ All are stored loaded with ammunition
2☐ Some are stored loaded with ammunition
1☐ None are stored loaded with ammunition

Willingness to discuss household firearm access with different individuals

- Asked of all respondents
- Willing was conceptualized as endorsement of “*somewhat willing*” or “*definitely willing*”
- Unwilling was conceptualized as endorsement of “*somewhat unwilling*” or “*definitely unwilling*”

56 Regardless of whether you have household firearms, how willing would you be to discuss your firearm access with each of the following individuals?

	Definitely willing	Somewhat willing	Somewhat unwilling	Definitely unwilling
A. Your primary care or mental health provider	4☐	3☐	2☐	1☐
B. A friend	4☐	3☐	2☐	1☐
C. A family member	4☐	3☐	2☐	1☐

Discussion(s) with healthcare provider(s) about firearm access

- Asked of all respondents

57 Have any of your healthcare providers (e.g., physician, nurse, psychologist, social worker) ever talked with you about your access to firearms?

1☐ Yes
2☐ No

Willingness to Alter Firearm Behaviors if Thinking about Suicide

- Asked of all respondents
- Willing was conceptualized as endorsement of “*somewhat willing*” or “*definitely willing*”
- Unwilling was conceptualized as endorsement of “*somewhat unwilling*” or “*definitely unwilling*”

58 If you were thinking about suicide, how willing would you be to:

	Definitely willing	Somewhat willing	Somewhat unwilling	Definitely unwilling
A. temporarily remove household firearms offsite?	4☐	3☐	2☐	1☐
B. temporarily remove ammunition for household firearms offsite?	4☐	3☐	2☐	1☐
C. unload and/or lock household firearms?	4☐	3☐	2☐	1☐

Perceptions of Firearms and Suicide Risk

- Asked of all respondents
- Agreement was operationalized as endorsement of "*Agree a little,*" "*Somewhat agree,*" "*Moderately agree,*" or "*Strongly agree*"
- Disagreement was operationalized as endorsement of "*Do not agree at all*"

59 We recognize that people's views about firearms vary widely. Please indicate how much you agree or disagree with each of the following statements.

	Strongly agree	Moderately agree	Somewhat agree	Agree a little	Do not agree at all
**A.** Having a firearm in the home increases the risk of dying by suicide.	1 ☐	2 ☐	3 ☐	4 ☐	5 ☐
**B.** Keeping firearms locked and unloaded can reduce the risk of dying by suicide.	1 ☐	2 ☐	3 ☐	4 ☐	5 ☐
**C.** Temporary removal of firearms from the home, such as legal offsite storage, can reduce the risk of dying by suicide.	1 ☐	2 ☐	3 ☐	4 ☐	5 ☐

Supplemental Table 1. *Characteristics of Veterans Overall (n=17,396)*

	N	%	95% CI
**Age [missing n=342]**			
18-34	660	9.67	8.80, 10.62
35-49	2,065	18.76	17.86, 19.70
50-64	4,782	29.86	28.97, 30.76
65-79	7,722	33.55	32.75, 34.36
80+	1,825	8.16	7.75, 8.58
**Sex Assigned at Birth [missing n=286]**			
Male	13,869	89.13	88.66, 89.59
Female	3,227	10.80	10.34, 11.27
Other	14	0.07	0.04, 0.14
**Gender [missing n=306]**			
Man	13,815	88.77	88.29, 89.24
Woman	3,180	10.62	10.17, 11.09
Transgender man	24	0.12	0.06, 0.21
Transgender woman	26	0.22	0.13, 0.35
Genderqueer/non-conforming	37	0.23	0.15, 0.36
Other	8	0.04	0.02, 0.11
**Sexual Orientation [missing n=612]**			
Heterosexual	15,955	95.26	94.80, 95.69
Gay/lesbian	393	1.99	1.75, 2.27
Bisexual	283	1.75	1.49, 2.07
Questioning	78	0.48	0.34, 0.66
Pansexual/omnisexual	14	0.15	0.08, 0.28
Asexual	17	0.14	0.06, 0.29
Other	44	0.23	0.16, 0.33
**Race/Ethnicity[1] [missing n=439]**			
White	14,018	85.41	84.83, 85.98
Black/African American	2,183	11.4	10.90, 11.91
Hispanic or Latine	1,565	7.90	7.36, 8.47
American Indian/Alaska Native	774	3.58	3.25, 3.94
Asian	457	2.33	2.05, 2.64
Native Hawaiian/Pacific Islander	124	0.62	0.47, 0.83
Other	141	0.88	0.63, 1.23
**Region of Residence**			
Midwest	3,710	20.52	19.92, 21.12
Northeast	2,627	12.82	12.33, 13.33
South	6,482	44.06	43.33, 44.80
West	4,341	22.21	21.60, 22.84
Puerto Rico	236	0.39	0.31, 0.49

**Rurality of Residence**			
Not rural	13,121	79.01	78.24, 79.76
Rural	4,275	20.99	20.24, 21.76
**Branch[1]**			
Army	7,883	43.98	42.99, 44.98
Air Force	4,064	22.66	21.82, 23.53
Navy	3,970	22.76	21.94, 23.60
Marines	1,649	11.32	10.61, 12.07
Coast Guard	273	1.50	1.29, 1.74
**Reserve/National Guard Branch[1] [missing n=20]**			
Army Reserve	1,879	10.36	9.78, 10.97
Army National Guard	1,274	7.61	7.06, 8.19
Air Force Reserve	704	3.80	3.44, 4.19
Air National Guard	411	2.29	2.00, 2.61
Navy Reserve	1,259	7.00	6.52, 7.51
Marine Corps Reserve	341	2.26	1.96, 2.61
Coast Guard Reserve	81	0.48	0.37, 0.62
**Ever Deployed [missing n=1,026]**			
Yes	11,729	72.25	71.30, 73.18
No	4,641	27.75	26.82, 28.70
**Service Era[1] [missing n=171]**			
Pre-Korean	188	0.89	0.76, 1.04
Korean War	656	2.83	2.59, 3.09
Post-Korean	2,137	9.50	9.06, 9.95
Vietnam War	7,519	33.31	32.51, 34.11
Post-Vietnam	7,054	37.83	36.91, 38.76
Desert Storm	4,857	31.27	30.33, 32.23
OEF/OIF/OND	3,629	30.95	29.96, 31.97
**VHA Enrollment [missing n=1,028]**			
Enrolled in VHA	9,737	54.82	53.79, 55.85
**Used VHA Services [missing n=579]**			
No, never	7,303	49.10	48.08, 50.13
Yes, in the past 12 months	7,355	37.49	36.52, 38.47
Yes, but not in the past 12 months	2,159	13.41	12.72, 14.18

Notes. Frequencies (n's) presented are unweighted, but percentages and CIs reflect weighted estimates. CI = confidence interval; OEF/OIF/OND = Operation Enduring Freedom, Operation Iraqi Freedom, and Operation New Dawn; VHA = Veterans Health Administration.
[1] Not mutually exclusive.

Supplemental Table 2. *Characteristics, by Veterans' Responses to the Household Firearm Access Response (n=17,134)*

	Yes (n=9,537)			No (n=6,461)			Decline to Respond (n=1,136)			Chi-sq	p-value
	N	%	95% CI	N	%	95% CI	N	%	95% CI		
**Age [missing n=289]**											
18-34	365	9.38	8.24, 10.66	227	8.88	7.46, 10.55	67	16.28	12.49, 20.95	66.10	<.0001
35-49	1,128	18.84	17.64, 20.11	750	17.82	16.35, 19.39	184	24.18	20.60, 28.16		
50-64	2,603	29.99	28.80, 31.21	1,781	29.56	28.09, 31.07	371	30.68	27.39, 34.18		
65-79	4,336	33.92	32.83, 35.03	2,862	34.51	33.11,35.94	420	24.55	21.90, 27.40		
80+	985	7.86	7.33, 8.43	730	9.23	8.50, 10.01	66	4.31	3.26, 5.68		
**Sex Assigned at Birth [missing n=215]**											
Male	7,906	90.74	90.14, 91.31	4,849	85.73	84.85, 86.57	934	91.21	89.41, 92.74	105.94	<.0001
Female	1,514	9.17	8.60, 9.77	1,521	14.22	13.39, 15.10	182	8.76	7.24, 10.56		
Other	8	0.09	0.04, 0.20	4	0.04	0.01, 0.15	1	0.03	0.00, 0.39		
**Gender [missing n=232]**											
Man	7,880	90.45	89.84, 91.03	4,832	85.25	84.36, 86.10	925	90.9	89.07, 92.45	97.62	<.0001
Woman	1,495	9.04	8.48, 9.64	1,495	13.89	13.07, 14.76	180	8.83	7.30, 10.65		
Transgender man	12	0.09	0.04, 0.18	12	0.19	0.08, 0.47	0				
Transgender woman	15	0.25	0.13, 0.46	8	0.19	0.08, 0.45	3	0.15	0.04, 0.61		
Genderqueer/non-conforming	11	0.13	0.06, 0.26	24	0.46	0.26, 0.80	2	0.04	0.00, 0.41		
Other	4	0.05	0.02, 0.18	3	0.02	0.00, 0.09	1	0.08	0.01, 0.49		
**Sexual Orientation [missing n=508]**											
Heterosexual	8,954	96.6	96.06, 97.06	5,811	92.69	91.71, 93.57	1,039	96.52	94.79, 97.70	72.51	<.0001
Gay/lesbian	145	1.30	1.05, 1.60	229	3.29	2.75, 3.93	16	1.47	0.80, 2.68		
Bisexual	133	1.36	1.06, 1.74	133	2.49	1.96, 3.14	17	1.67	0.88, 3.15		
Questioning	28	0.34	0.19, 0.60	43	0.76	0.51, 1.13	6	0.27	0.09, 0.81		
Pansexual/omnisexual	8	0.16	0.07, 0.35	5	0.15	0.05, 0.47	1	0.06	0.01, 0.47		
Asexual	7	0.08	0.03, 0.20	10	0.27	0.10, 0.72	0				
Other	16	0.18	0.10, 0.30	25	0.36	0.22, 0.59	0				

**Race/Ethnicity[1] [missing n=427]**											
White	8,050	88.63	87.82, 89.39	4,944	81.08	79.88, 82.34	878	83.38	79.98, 86.29	84.46	<.0001
Black/African American	988	9.38	8.71, 10.10	1,031	14.79	13.76, 15.88	141	11.94	9.66, 14.68	57.80	<.0001
Hispanic or Latine	662	6.25	5.59, 6.99	804	11.38	10.27, 12.60	81	4.99	3.69, 6.72	75.42	<.0001
American Indian/Alaska Native	445	3.64	3.19, 4.15	247	3.22	2.71, 3.82	72	4.69	3.49, 6.27	4.48	0.1063
Asian	192	1.62	1.31, 1.99	226	3.40	2.87, 4.03	34	3.18	1.93, 5.22	24.57	<.0001
Native Hawaiian or Other Pacific Islander	55	0.46	0.32, 0.66	60	0.89	0.55, 1.43	9	0.75	0.33, 1.68	5.37	0.0682
Other	70	0.70	0.40, 1.23	54	1.02	0.70, 1.48	17	1.76	0.69, 4.42	3.46	0.1778
**Region of Residence**											
Midwest	2,137	21.50	20.67, 22.35	1,275	18.91	17.84, 20.03	240	19.64	16.68, 22.97	160.66	<.0001
Northeast	1,197	10.50	9.88, 11.15	1,249	17.13	16.18, 18.11	147	11.75	9.55, 14.37		
South	3,778	47.12	46.10, 48.14	2,143	38.35	36.94, 39.78	445	45.24	41.39, 49.15		
West	2,390	20.74	19.88, 21.64	1,599	24.73	23.43, 26.08	299	23.3	19.94, 27.04		
Puerto Rico	35	0.15	0.08, 0.28	195	0.88	0.68, 1.14	5	0.08	0.01, 0.48		
**Rurality of Residence**											
Not rural	6,727	75.17	74.06, 76.24	5,413	86.50	85.36, 87.57	813	76.72	73.48, 79.67	164.42	<.0001
Rural	2,810	24.83	23.76, 25.94	1,048	13.50	12.43, 14.64	323	23.28	20.33, 26.53		
**Branch (Ever Served)[1]**											
Army	4,373	44.81	43.47, 46.16	2,882	42.11	40.48, 43.75	489	44.35	40.42, 48.37	5.56	0.062
Air Force	2,200	21.93	20.84, 23.06	1,526	23.69	22.23, 25.22	286	24.05	20.67, 27.78	3.76	0.1524
Navy	2,082	21.9	20.81, 23.03	1,583	24.87	23.48, 26.33	254	20.22	17.31, 23.47	12.63	0.0018
Marines	1,001	12.45	11.49, 13.48	509	9.39	8.29, 10.63	112	11.65	9.21, 14.64	14.02	0.0009
Coast Guard	143	1.38	1.12, 1.70	107	1.74	1.37, 2.20	21	1.50	0.87, 2.56	2.06	0.3569
**Reserve/National Guard Branch (Ever Served)[1] [missing n=19]**											
Army Reserve	1,060	10.96	10.16, 11.82	693	9.73	8.82, 10.72	105	8.87	6.99, 11.18	5.39	0.0674
Army National Guard	770	8.32	7.58, 9.13	382	6.08	5.23, 7.06	105	9.18	7.25, 11.57	15.01	0.0005
Air Force Reserve	386	3.67	3.21, 4.19	266	3.81	3.27, 4.44	44	4.64	2.89, 7.38	1.19	0.551
Air National Guard	240	2.54	2.13, 3.02	137	1.95	1.56, 2.44	33	2.07	1.36, 3.13	3.90	0.142

Navy Reserve	685	7.1	6.46, 7.81	492	7.18	6.44, 7.98	70	5.81	4.08, 8.22	1.61	0.4474
Marine Corps Reserve	200	2.34	1.96, 2.78	113	2.23	1.69, 2.94	23	2.00	1.27, 3.14	0.3438	0.842
Coast Guard Reserve	42	0.43	0.29, 0.63	35	0.61	0.42, 0.89	4	0.30	0.09, 0.98	2.26	0.3222
**Ever Deployed [missing n=947]**											
Yes	6,699	74.27	73.04, 75.47	4,091	68.25	66.56, 69.89	784	73.29	69.63, 76.65	31.93	<.0001
No	2,355	25.73	24.53, 26.96	1,952	31.75	30.11, 33.44	306	26.71	23.35, 30.37		
**Service Era (Ever)[1] [missing n=148**											
Pre-Korean	85	0.72	0.57, 0.91	86	1.19	0.94, 1.51	7	0.55	0.26, 1.18	10.75	0.0046
Korean War	334	2.58	2.28, 2.92	277	3.51	3.07, 4.01	23	1.13	0.65, 1.93	26.92	<.0001
Post-Korean	1,234	9.83	9.24, 10.46	767	9.68	8.93, 10.48	83	5.00	3.87, 6.43	30.80	<.0001
Vietnam War	4,271	33.98	32.90, 35.08	2,718	33.62	32.24, 35.03	413	24.77	22.11, 27.65	32.17	<.0001
Post-Vietnam	3,866	38.35	37.11, 39.61	2,653	37.92	36.38, 39.49	465	33.7	30.34, 37.24	6.20	0.0451
Desert Storm	2,700	31.94	30.68, 33.22	1,749	30.17	28.62, 31.77	373	33.18	29.56, 37.00	3.65	0.1614
OEF/OIF/OND	2,028	30.94	29.58, 32.34	1,249	28.52	26.77, 30.34	336	45.08	40.93, 49.29	55.39	<.0001
**VHA Enrollment [missing n=937]**											
Enrolled in VHA	5,442	55.24	53.86, 56.62	3,494	52.29	50.57, 54.01	673	61.16	57.11, 65.05	17.74	0.0001
**Used VHA Services [missing n=479]**											
No, never	4,044	49.60	48.24, 50.97	2,791	50.01	48.31, 51.71	422	42.64	38.57, 46.82	14.07	0.0071
Yes, in the past 12 months	4,095	37.51	36.21, 38.82	2,644	36.20	35.60, 37.84	519	41.25	37.29, 45.32		
Yes, but not in the past 12 months	1,178	12.89	12.01, 13.82	813	13.78	12.60, 15.06	149	16.11	13.20, 19.51		

Notes. Frequencies (n's) presented are unweighted, but percentages and CIs reflect weighted estimates. Some categories (for gender and sexual orientation) were collapsed for chi-square analyses.
CI = confidence interval; OEF/OIF/OND = Operation Enduring Freedom, Operation Iraqi Freedom, and Operation New Dawn; VHA = Veterans Health Administration.
[1] Not mutually exclusive.

Supplemental Figure 1. *Visual Displays Comparing Perceptions of Firearms and Suicide Risk, Willingness to Alter and Discuss Firearm Access, and Prior Firearms Discussions, Stratified based on Responses to the Household Firearm Access Question*

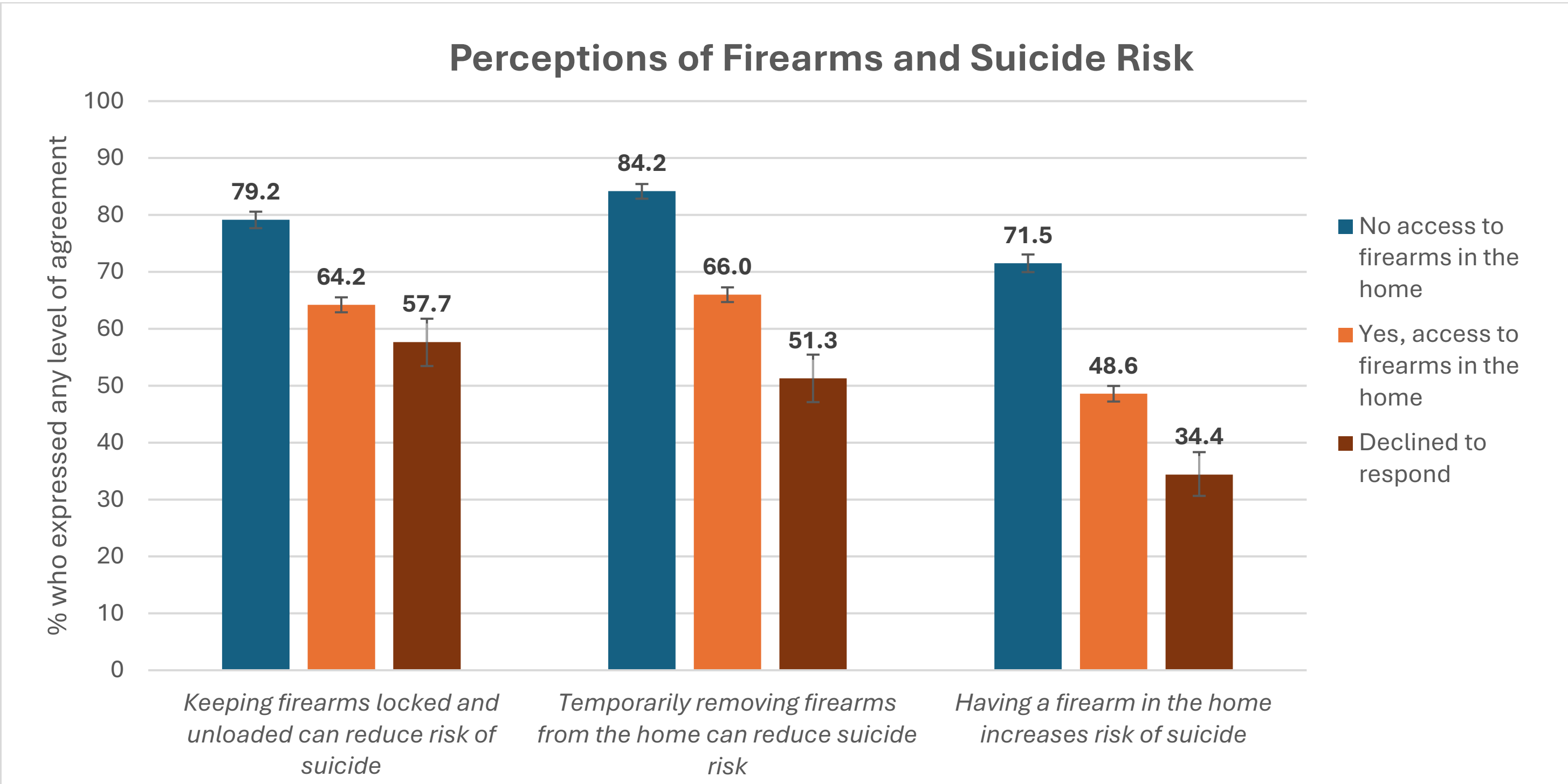

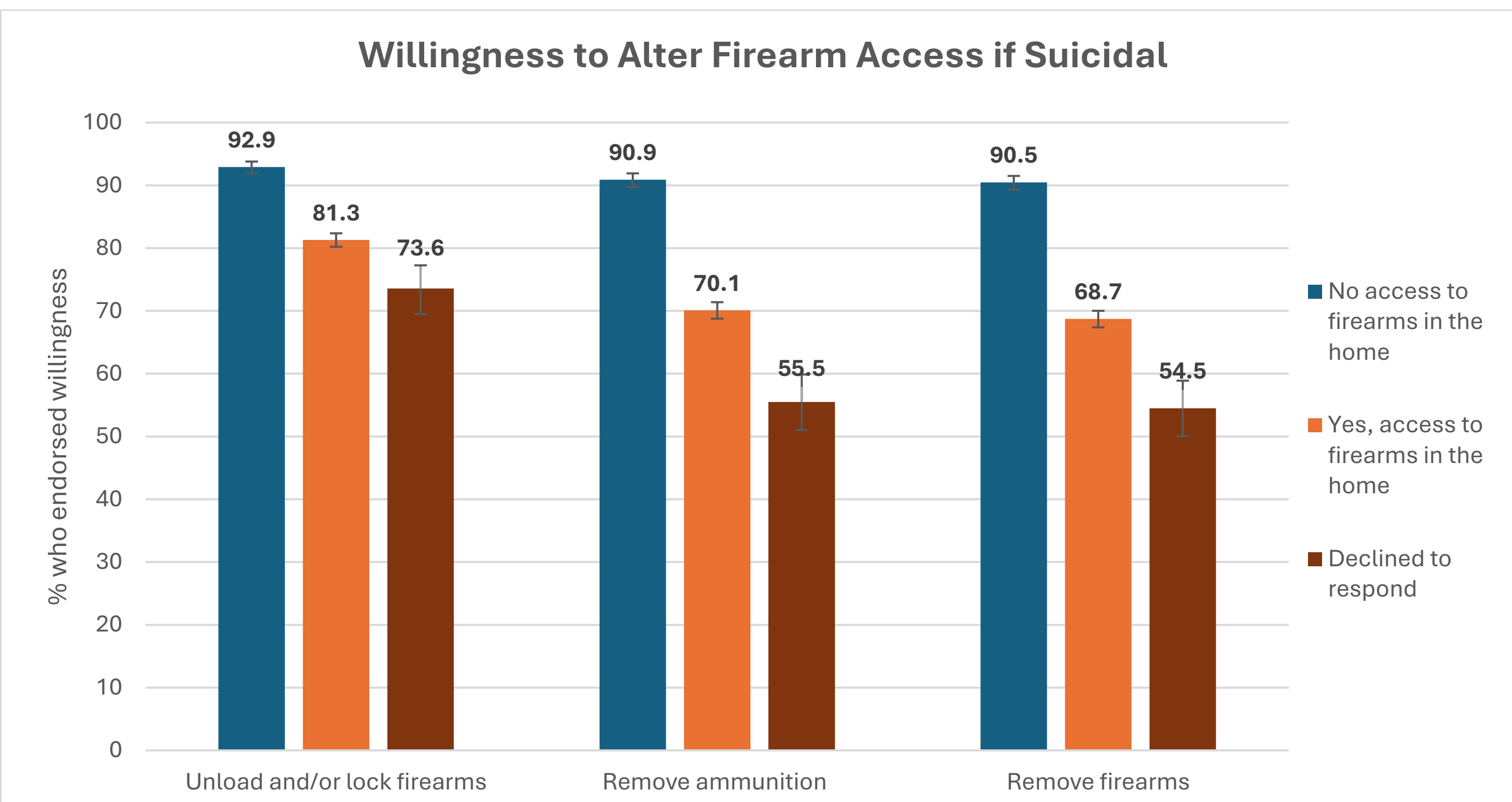

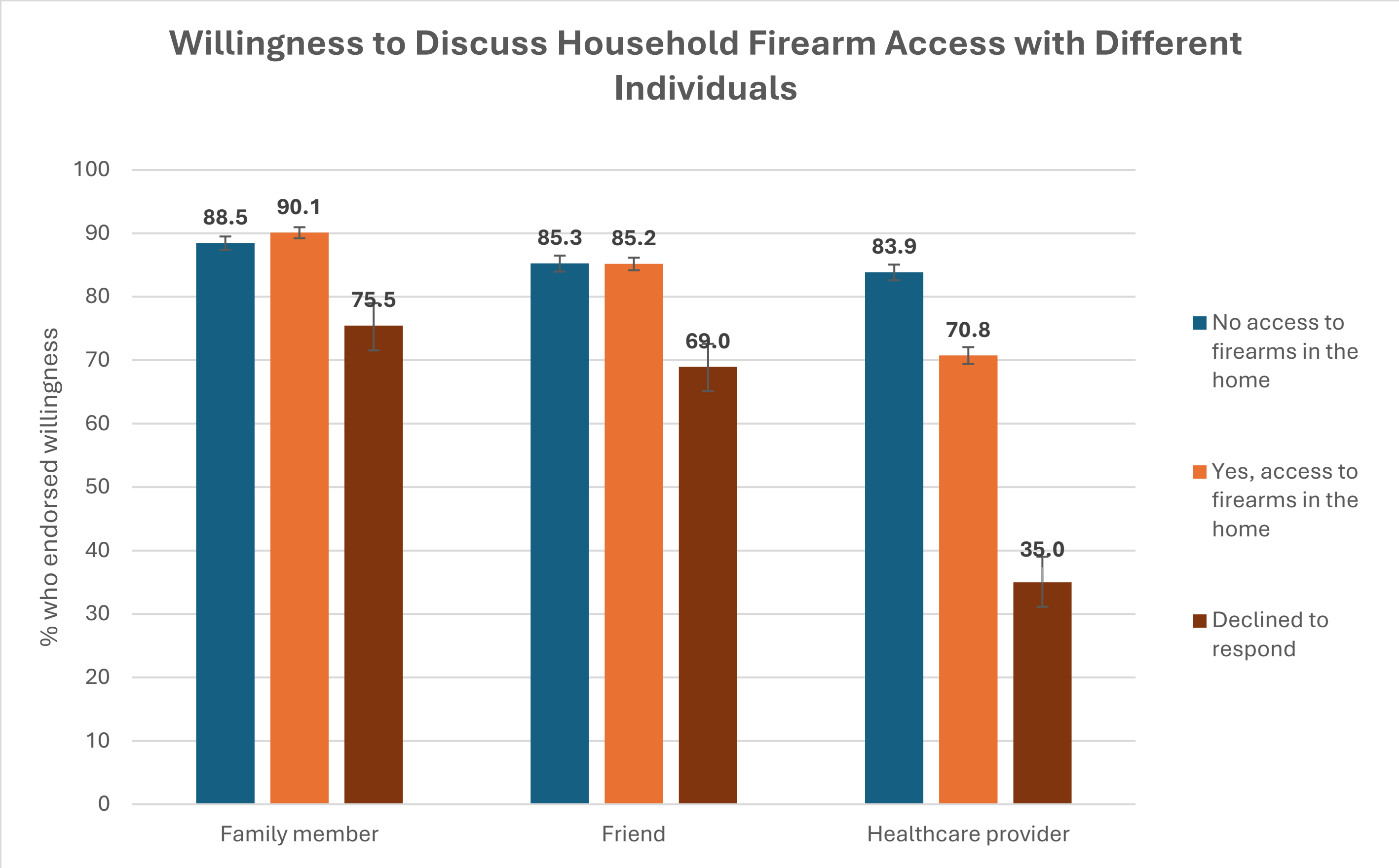
Willingness to Discuss Household Firearm Access with Different Individuals
100
90
80
70
60
50
40
30
20
10
0
% who endorsed willingness
88.5
90.1
75.5
85.3
85.2
69.0
83.9
70.8
35.0
Family member
Friend
Healthcare provider
No access to firearms in the home
Yes, access to firearms in the home
Declined to respond

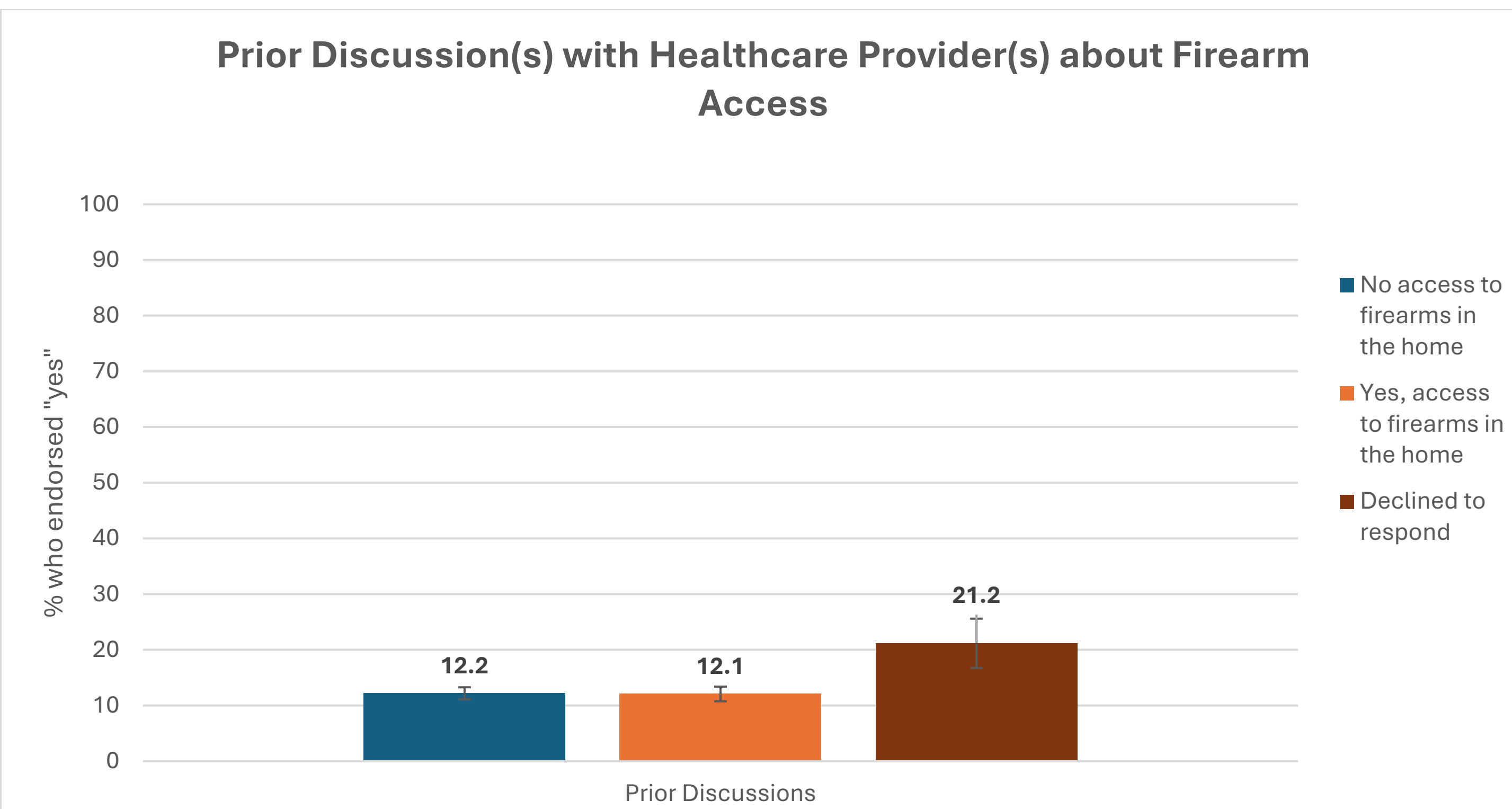
Prior Discussion(s) with Healthcare Provider(s) about Firearm Access
100
90
80
70
60
50
40
30
20
10
0
% who endorsed "yes"
12.2
12.1
21.2
Prior Discussions
No access to firearms in the home
Yes, access to firearms in the home
Declined to respond
